# Recurrence of Dislocation Following Total Hip Arthroplasty Revision Using Dual Mobility Cups Was Rare in 180 Hips Followed Over 7 Years

**DOI:** 10.1007/s11420-012-9301-0

**Published:** 2012-09-19

**Authors:** Patrice Mertl, Antoine Combes, Frédérique Leiber-Wackenheim, Michel Henri Fessy, Julien Girard, Henri Migaud

**Affiliations:** 1 Orthopaedics Department, University of Amiens, place Victor-Pauchet, 80054 Amiens, France; 2 Roger Salengro Hospital, Centre Hospitalier Régional Universitaire de Lille, 2 avenue Oscar Lambret, 59037 Lille Cedex, France; 3 Department of Orthopaedics, Traumatology and Sports Medicine, Centre Hospitalier Lyon Sud, chemin du Grand-Revoyet, 69495 Pierre Bénite Cedex, France; 4 Orthopaedics Department, University of Lille, 2 avenue Oscar Lambret, 59037 Lille Cedex, France

**Keywords:** hip arthroplasty, instability, dual mobility, revision, bearing, wear, polyethylene

## Abstract

**Background:**

Dual mobility (DM) cups of mobile polyethylene were introduced to prevent total hip arthroplasty (THA) dislocation, but no large series with this design to treat recurrent instability have been reported.

**Purpose:**

Our retrospective investigation ascertained the efficiency of DM cups in correction of recurrent dislocation and assessed any adverse effects.

**Methods:**

One hundred eighty THAs with recurrent instability were revised to DM cups in 180 patients (mean age, 67.4 ± 11.7 years; range, 19 to 92 years). Thirty-one patients (17.2%) underwent at least one earlier THA revision, and 15 (10.3%) incurred non-union of the greater trochanter. Of the initial group in 2009, 145 patients had completed evaluations which included assessment of the Harris Hip Score and a radiographic assessment at a mean follow-up of 7.7 ± 2.2 years (range, 4 to 14 years). The rate of survival was calculated considering any reason for revision as failure.

**Results:**

At follow-up, Harris hip score was 83.9 ± 16.1 (range, 21 to 100). Dislocation of the large articulation occurred in seven hips (4.8%), and only two recurred (1.4%) (one requiring additional revision). In addition, two intra-prosthetic dislocations of the small articulation (1.4%) were observed and needed revision surgery. The large number of earlier surgeries and non-union of the greater trochanter were related to recurrent instability. Two cups (1.4%) showed signs of definite loosening; six (4.1%) presented signs of possible loosening. Twenty-nine hips manifested femoral or acetabular osteolysis (20%), but only three were severe. Eight-year survival rate considering revision for any reason was 92.6% (95% CI, 85.5–96.4%).

**Conclusions:**

This series indicates that DM cups are a viable option to treat recurrent THA instability. Their design provides a low risk of recurrent instability without increasing mechanical complications.

## Introduction

Managing recurrent total hip arthroplasty (THA) instability is a difficult challenge because most of these patients are elderly and have undergone multiple earlier procedures, resulting in muscular lesions that compromise the stability of subsequent revision(s) [22, 24]. The reasons for recurrent instability are many and include component mal-orientation, inadequate restoration of leg length or the abductor lever arm, impingement, and muscular lesions. No consensus exists for the treatment of this complication as evidenced by the number of procedures recommended for correction of the problem [15, 22, 23, 35]. Current methods of dealing with recurrent instability have not proven to be significantly effective in large series, and none of them is considered to be the gold standard [22, 35, 37]. Among these procedures, use of constrained or tripolar cups is deemed to be successful but can be associated with a high rate of mechanical complications [15, 28] requiring revision with rates ranging from 2.3% to 20% [2, 3, 5, 7, 10, 36].

**Fig. 1:**
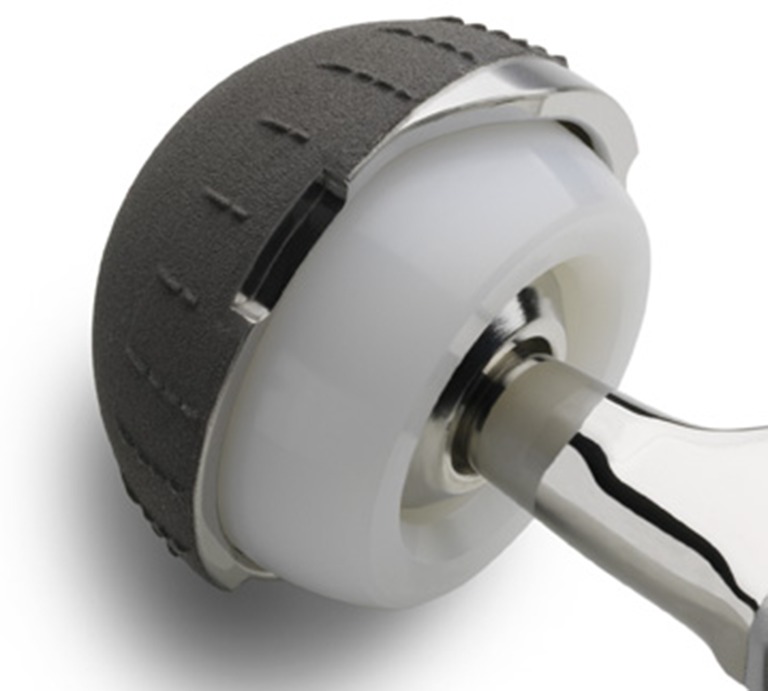
DM cup: The mobile polyethylene insert is not constrained in the metal back constituting the large articulation. It is clipped over the femoral head (22- or 28-mm diameter) constituting the small articulation. *Note* the smooth aspect of the head–neck junction as well as the absence of an extraction hole

Dual mobility (DM) cups (Fig. 1) were designed to prevent THA dislocation based on the large-diameter head concept. The polyethylene insert is freely mobile with respect to the metallic cup in a large-diameter articulation and coupled but mobile in relation to the conventional 22 or 28 mm femoral head at the small articulation. The design presently serves to manage recurrent THA instability, but no large series have reported results with DM cups for the indication of restoring stability to a THA with a history of recurrent dislocation.

The goals of the current retrospective, multicenter study were: (1) to ascertain the efficiency of DM cup design to prevent recurrent dislocation, (2) to assess the clinical function of these revised hips using the Merle d’Aubigné and Harris Hip Scores, and (3) to analyze survivorship as well as adverse effects that were observed including loosening and osteolysis.

## Materials and Methods

All ethics boards of the institutions involved in this multicenter study gave their approval. From 1995 to 2003, ten centers managed recurrent THA instability by revision with 180 DM cups. The latter were inserted in 180 patients (108 women and 72 men) with a mean age of 67.4 ± 11.7 years (range, 19 to 92 years). These 180 patients were assessed retrospectively in 2009. Of the initial group, 21 patients died (after a mean of 2.2 years, 6 months to 3.6 years), and 14 were lost to follow-up (after a mean of 1.8 years, range 2 months to 2.9 years), leaving 145 patients with complete clinical and radiological analyses in 2009. Mean body mass index (BMI) was 27.7 ± 4.9 (range, 15.4 to 59.2), and 44 patients (24%) were overweight with BMI >30 kg/m^2^. The initial diagnosis was primary osteoarthritis in 129 hips (71.6%), femoral head osteonecrosis in 19 hips (10.5%), arthritis secondary to hip dysplasia in ten hips (5.5%), and replacement because of various etiologies (inflammatory arthritis, post-infection, post-trauma) in 22 hips (12.2%). Thirty-two hips (17.7%) had at least one earlier THA revision, 16 hips had two earlier THA revisions, ten hips had three earlier THA revisions, and, finally, four hips and one hip respectively had four and five earlier THA revisions. Non-union of the greater trochanter was seen in 15 hips (8.3%) at the time of DM insertion. The time period from the earlier THA to the index procedure was 43 months (range, 1 to 228 months). Before the index procedure, 88 patients were rated according to Charnley’s categorization [8] as class A (one hip involved), 50 as class B (both hips involved), and 30 as class C (multi-articular disease) (the status of 12 patients was unknown). Likewise, according to Devane scores [11], the majority of patients were not active—seven were rated as grade 5 (heavy profession and/or contact sports), 23 as grade 4 (light job and/or noncontact sports), 51 as grade 3 (leisure activities), 67 as grade 2 (semi-sedentary), and 19 as grade 1 (sedentary) (the status of 13 patients was unknown).

All patients received hemispherical DM cups with a 3-mm-thick metal shell and a mobile polyethylene insert (mobile with respect to the metal back) (Fig. 1). The polyethylene insert was hemispherical, of ultra-high molecular weight, variable in thickness, but always greater than or equal to 6 mm (variable according to cup and femoral head diameter). Polyethylene insert concavity was articulated with the femoral head after its impaction by force over a polyethylene rim designed for retention (Fig. 1). Its design theoretically reduces the risk of dislocation according to two principles: The mobile insert prevents prosthetic neck impingement over the polyethylene rim, and the large articulation between the insert and the metal back increases range of motion before dislocation. At the time of insertion, unstable THAs were revised without cup loosening in 119 hips. In the remaining 61 hips, the cups were loose with varying degrees of bone loss judged according to the criteria proposed by Paprosky and Burnett [34]. Forty-six hips were assessed as grade 1; 12 hips were judged as grade 2 and two hips as grade 3.

**Fig. 2:**
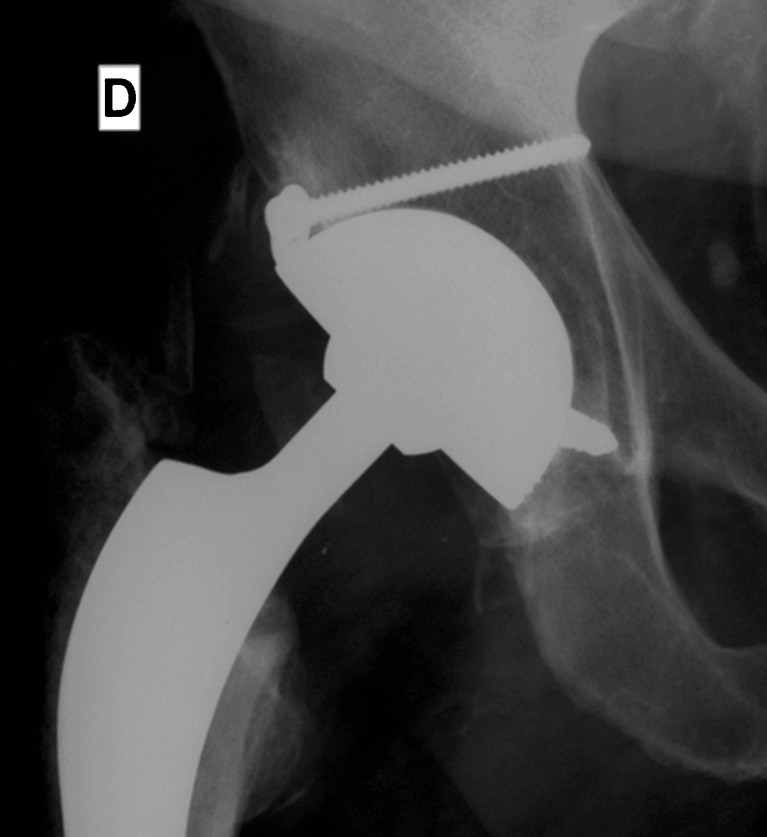
Cementless DM cup with additional screw and pegs for treating recurrent dislocation

Cup fixation was cementless in 159 hips (88%), while 21 cups (12%) were cemented because of insufficient primary fixation or severe bone loss (eight of these cups were cemented in a cage and 13 were cemented into the pelvic bone). Additional acetabular, morselized bone allografting was undertaken in 21 hips. Cementless fixation (Fig. 2) was achieved in 159 hips with porous hydroxyapatite coating, with press fit alone in 40 hips (22.2%), additional screws in 20 hips (11.1%), and screws plus pegs in 99 hips (55.5%) (Fig. 2). The femoral head was cobalt-chrome in 165 hips (91.7%) and alumina ceramic in 15 hips (8.3%). During the index procedure, the stem was left in place in 151 cases (83.9%) and needed revision because of malpositioning or loosening in 29 (16.1%). The surgical approach was postero-lateral in 149 hips (82.8%), lateral transgluteal in 29 hips (16.1%), and lateral with trochanteric osteotomy in two hips (1.1%).

Full weight bearing was permitted the day after surgery in 160 hips (88.9%) but not for 20 of 21 hips that underwent previously mentioned acetabular bone grafting; these hips were allowed full weight bearing after a mean of 6 weeks (4–12 weeks) following the index procedure.

**Fig. 3:**
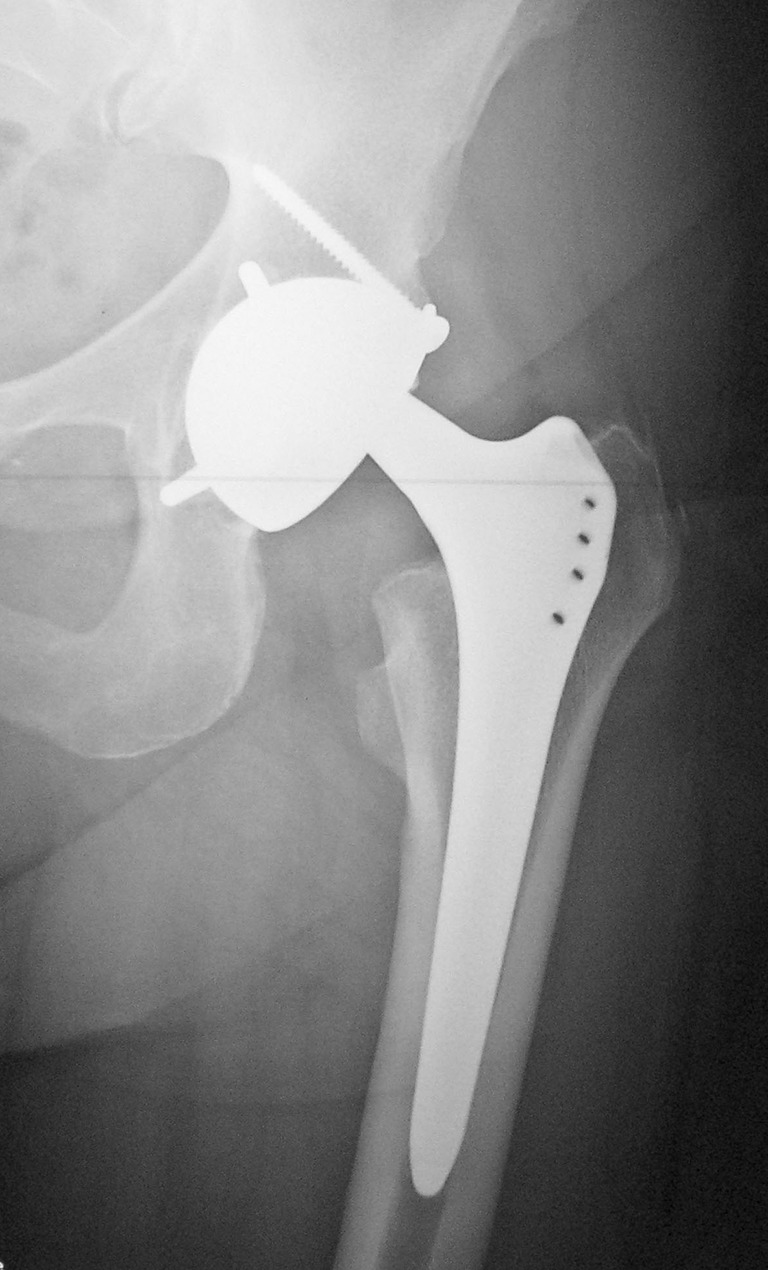
Intra-prosthetic dislocation 5 years after revision of recurrent instability. The stem neck is not fully polished, which could favor polyethylene rim wear and dislocation of the small articulation. Cup exchange was needed as long as patient was referred 2 weeks after dislocation with lesions on the articulating inner wall of the metal back

An observer was selected for each of the ten study centers to assess patients according to the same criteria. The ten observers chosen did not participate in the surgeries and were required to appraise patients in their corresponding center in 2009. Clinical evaluation was conducted according to the Merle d’Aubigné hip score [31] and the Harris hip score [19], with the hips being pooled according to Charnley’s classification [8]. Activity level was ascertained according to Devane et al. [11]. X-rays were taken at follow-up and compared with post-operative X-rays to examine cup fixation according to Massin et al. [30]. Heterotopic ossification was rated according to Brooker et al. [6]. Acetabular inclination was measured by angle with respect to U landmarks. The dislocation rate (with large articulations) was recorded as early dislocations (up to 3 months after revision to DM cups) or late dislocations (over 3 months after revision to DM cups). The intra-prosthetic dislocation rate was recorded in the same manner. Intra-prosthetic dislocation is a specific complication of DM cups (Fig. 3). The head extracts from the polyethylene rim, indicating failure of retention between the head and polyethylene in small articulations (allowing the femoral head to articulate directly with the metal back).

The results were analyzed in 145 patients (145 hips) who underwent complete clinical and radiological assessment in 2009. Categorical variables were expressed as frequency and percentage and numerical variables as mean, standard deviation, and range. Percentages were compared by the chi-square test or Fisher’s exact test. The evolution of functional scores was analyzed by the Wilcoxon test (comparison of distribution). Survivorship was measured according to the Kaplan–Meier test with three types of endpoints: (a) cup revision for any reason, (b) cup revision because of fixation failure or polyethylene insert exchange with intra-prosthetic dislocation or recurrent dislocation, and (c) cup revision for fixation failure. The 95% confidence intervals (95% CI) were reported. Statistical analysis was performed with SAS™ version 9.1 (SAS Institute, Inc., Campus Drive, Cary, NC, USA). The level of significance was set at *p* = 0.05.

## Results

The DM cup was successful in preventing recurrent dislocation in the majority of these THAs. Only seven hips (4.8%) suffered dislocation of the large articulation, and only two recurred (1.4%) (with one needing repeated revision). In addition, two intra-prosthetic dislocations of the small articulation (1.4%) were observed and required revision surgery (Fig. 3). A larger number of prior THA revisions and non-union of the greater trochanter were correlated with recurrent instability (*p* < 0.05). Four hips had dislocation among the 15 hips that had non-union of the greater trochanter (26.6%) versus three of the 130 hips that had intact greater trochanter (2.3%) (*p* = 0.002; *F* exact test). Likewise, the number of former THA revisions was 1.85 among recurrently unstable hips versus 1.2 in those without recurrence (Mann–Whitney’s test, *p* = 0.002). The small number of events did not allow us to identify a reliable cutoff using a ROC curve analysis. Nevertheless, we computed the odds ratio between the groups of patients without previous THA revisions versus those with at least one THA revision. The value of the odds ratio was 14.7 (95% CI, 2.7–79.9, *p* = 0.002), indicating a higher risk of recurrent dislocation when the hip had multiple former THA revisions. On the other hand, cup inclination was not related to recurrence of dislocation. Mean inclination was 44.4° (range, 40° to 60°) in the group that suffered recurrent dislocation, versus 50° (range, 10° to 80°) in those without recurrence (*p* = 0.7).

The Harris hip score increased from a mean of 76.9 ± 23.1 (range, 4 to 100) to 83.9 ± 16.1 (range, 21 to 100). The Merle d’Aubigné functional score rose from 14.1 ± 3.8 (range, 2 to 18) to 15.7 ± 2.5 (range, 7 to 18). One hundred twenty-seven hips (87.6%) had no pain or rare or mild pain. In particular, according to Merle d’Aubigné’s hip rating, mean pain score climbed from 4.7 ± 1.8 (range, 0 to 6) to 5.3 ± 1.1 (range, 1 to 6), mean mobility score, from 5.4 ± 1.1 (range, 0 to 6) to 5.7 ± 0.7 (range, 2 to 6), and mean walking score, from 4.1 ± 1.8 (range, 0 to 6) to 4.7 ± 1.6 (range, 0 to 6). According to Charnley’s classification [8], at follow-up, 53 patients (36.5%) were rated as grade A, 41 patients (28.3%) as grade B, and 51 patients (35.2%) as grade C. According to the Devane’s scoring system [11], patients had lower activity scores than before the index procedure: at follow-up, 2 (1.4%) were rated as grade V, 11 (7,6%) as grade IV, 69 (47.6%) as grade III, 48 (33.1%) as grade II, and 15 (10.3%) as grade I.

The rate of complications appears to be low in this cohort. Forty-five cases of heterotopic ossifications were observed, rated according to Brooker et al. [6] as grade I in 36 hips, grade II in seven hips, and grade III in two hips. Mean cup inclination was 49.4° ± 10.1 (range, 10° to 80°). Ten (6.8%) revisions were repeated for two cup loosenings, one peri-prosthetic femoral fracture, four infections, two intra-prosthetic dislocations, and one recurrent instability. Only five (3.4%) revisions were directly related to DM cups (two cup loosenings and three dislocations, of which two were intra-prosthetic). Only two cups (1.4%) showed signs of definite loosening (both were revised as previously mentioned). Six (4.1%) presented signs of possible loosening. In contrast, 29 (20%) manifested osteolysis (14 around the cup, 18 at the femur, 3 were combined femoral and acetabular), but only three were extensive. Osteolysis was more frequent when the femoral neck was cylindrical instead of flat and rough instead of polished (*p* < 0.01) (Table 1). The 8-year survival rate considering revision for any reason was 92.6% (95% CI, 85.5–96.4%). For cup revision (fixation failure or polyethylene insert exchange), survivorship was 96.2% (95% CI, 90–98%), and survivorship assessing for cup fixation failure was 97.5% (95% CI, 92.2–99.2%)

**Table 1:** Rate of osteolysis according to the shape and surface finish of the femoral neck in 145 hips at follow-up

		Osteolysis	No osteolysis	Significance
Shape of femoral neck (*n* = 109)^a^	Flat	3 (10.3%)	26	*P* = 0.01
	Cylindrical	15 (18.7%)	65	
Surface finish of femoral neck (*n* = 119)^a^	Polish	2 (4.2%)	47	*P* = 0.004
	Rough	15 (21.4%)	55	

^a^Values are missing in 36 hips regarding femoral shape (11 of these had osteolysis) and in 26 hips regarding surface finish (12 of these had osteolysis)

## Discussion

Surgical management of recurrent total hip instability is a demanding procedure. Tripolar or constrained cups, currently recommended for this indication, give an acceptable rate of recurrent dislocation but at the cost of revisions related to mechanical complexity [2, 3, 7, 10, 32, 36, 37]. Using DM cups for the same indication, few studies are reporting a low rate of recurrent dislocation (ranging from 1.7% to 5.5%), but these were based on populations of limited size [16, 18, 27]. We therefore designed the current study based on a large population to accurately assess the efficiency of DM cups to prevent recurrent dislocation as well as clinical function, potential adverse effects (loosening and osteolysis), and survivorship. The main finding in this large cohort was the low rate of recurrent dislocation (4.8%) with DM cups to revise THA instability. It represented a non-selected population with a high rate of earlier THA revisions (17.7%) and trochanter non-union (8.3%), considered to be factors promoting recurrent instability [7, 22, 35]. In addition, two intra-prosthetic dislocations of the small articulation (1.4%) required revision surgery. In summary, the rate of instability (large and small articulations) was 6.2% (nine cases), but only three (2%) needed revision surgery.

Our series had some limitations due to its retrospective nature and lack of a control group. In addition, almost 6.7% of patients died, and 11.7% were lost to follow-up, but the majority remained for minimum 1-year follow-up, allowing short-term assessment of recurrent dislocation. On the other hand, the large patient population with recurrent instability after THA strengthened the conclusions and gave us an opportunity to measure the efficiency of DM cups in preventing dislocation in revision. Moreover, most revisions in the current study were limited to cup exchange (151 cases, 83.8%), allowing us to estimate the isolated effect of DM cups in managing recurrent THA dislocation.

The rate of recurrent instability in this study is higher than that reported, also with DM cups, by Guyen et al. [16] (5.5% at a mean of 4 years), Leiber-Wackenheim et al. [27] (1.7% at a mean of 8 years), and Hamadouche et al. [18] (4% at a mean of 4.2 years). In contrast, the current series was larger (almost double), had a higher number of former THA revisions and a higher proportion of greater trochanter non-union. In addition, the current rate of recurrent dislocation requiring repeated revision (1.4%) was similar to that observed by Guyen et al. [16] and Leiber-Wackenheim et al. [27]. These results were obtained without an increase in adverse effects involving mechanical failure. In total, the two cup loosenings (1.4%) (both revised) were much lower than with constrained or tripolar cups for the same indication [7, 36]. Particular attention should be paid when DM cups are inserted to treat unstable THAs that have undergone multiple earlier revisions or in case of greater trochanter non-union. The current study underlines DM cups have a higher rate of failure in stabilizing the THA in these situations. This last result advocates for the repair of greater trochanter at the time of revision and may suggest the use of constrained cups despite that this design has no specific report for these particular indications [2, 3, 5, 7, 10, 28, 36].

The surgical treatment of recurrent dislocation after THA varies between surgeons, institutions, and countries. Saadat et al. [35] underscored the need to explore etiologies—implant malorientation, insufficient soft-tissue tension, the cam effect—which should be corrected, if found. However, studies of revision for etiological correction have delivered poor results with recurrent dislocations ranging from 24% to 39% [9, 12]. Revision by wedge augmentation or liner exchange also carries an elevated risk of recurrence reported to be between 17% and 24% [29, 33]. Bidar et al. suggest these procedures should be restricted to non-loose and correctly oriented cups [4]. Revision of recurrent dislocation using implants with increased stability has the advantage of correcting some etiological factors, such as malorientation, while enhancing mechanical resistance to dislocation. Large bearing diameters were advocated to prevent dislocation [20]. Sikes et al. [38] and Amstutz et al. [1] recommended large-diameter cups (metal-on-metal or metal-on-polyethylene) but recorded recurrence rates greater than 14%. In addition, changes in friction torque expose patients to specific complications including metallic debris dissemination from metal-metal THA [21], and breakage and squeaking from alumina-alumina THA [17]. Likewise, large-diameter heads with highly cross-linked polyethylene have limitations concerning polyethylene thickness as well as osteolysis related to small particle size [32]. Constrained tripolar cups have been proposed to manage THA instability [13]. With such a design, Beaulé et al. [2] reported 9.5% recurrence, Dela Valle et al. [10] 20%, Berend et al. [3] 28.9%, and Bremner et al. [5] 7%. Using these components, late revisions occur due to mechanical failures (ranging from 2.3% to 20%) (Table 2) [2, 3, 5, 7, 10].

**Table 2:** Outcome of treatment of recurrent unstable hips revised by constrained and tripolar cup versus the DM cups in the current series

Authors	Number of hips	Implant	Mean follow-up (years)	Recurrence of dislocation or failure of constrain mechanism	Revision for dislocation or failure of constrain mechanism
Beaulé et al. [2]	21	Tripolar constrained	5.4	9.5%	9.5%
Berend et al. [3]	128	Constrained liner	10	28.9%	–
Bremner et al. [5]	56	Tripolar constrained	10.6	7%	5.3%
Carter et al. [7]	59	Constrained liner	5.5	20%	20%
Della Valle et al. [10]	41	Constrained liner	3.6	20%	20%
Shapiro et al. [36]	87	Tripolar constrained	4.8	2.3%	2.3%
Current series	180	Dual mobility	7.7	6.2%	2.1%

In former studies as well as in the current one, DM cups guarantee clinical results and survivorship similar to modern THAs with a low rate of revision related to mechanical failure (2.1%) [16, 18, 23, 27]. DM cups simplify revisions, as they do not require femoral exchange, being compatible with 28- and 22.2-mm femoral heads. In addition, they can be fixed by cement to cages when acetabular reconstructions are necessary during revision related to recurrent instability, giving an opportunity to improve hip centering and subsequently downsize the risk of instability [18]. One of the limitations of DM cups is the risk of intra-prosthetic dislocation [26]. The rate of this specific and rare complication is below 1% and was reported as 0% for Leclercq et al. [25] at 5-year follow-up, 3.7% for Guyen et al. [14], and 0% for Leiber-Wackenheim et al. [27]). Whenever intra-prosthetic dislocation occurs, it requires revision that is usually a simple liner exchange, unless damage to the metal back’s inner face warrants cup exchange. It is possible that this can be prevented by head–neck-junction designs that are smooth because the neck behaves as a third articulation [25–27]. In the current study, 83.9% of stems were not revised, exposing the polyethylene rim to old head–neck junctions that were not fully compatible (round, polished, without extraction hole) with DM cups (Fig. 3). Similarly, the shape and finish of the head–neck-junction was incriminated in osteolysis occurrence that was rare (20%) and mainly not extensive (Table 1). This last result confirms that the neck behaves as a third articulation with regard to mobile polyethylene insert [25–27].

Our series indicates that DM cups are a viable option to treat recurrent THA instability. The design provides a low risk of recurrent instability without increasing mechanical complications, particularly when compared with constrained or tripolar cups. DM cups have the advantage of decreasing the need for stem exchange in complex revisions, as they can be used with 22- or 28-mm heads, especially if the head–neck junction is properly designed to interact with DM components. Particular attention should be paid when inserting DM cups after multiple earlier THA revisions or in case of greater trochanter non-union. These encouraging outcomes should be confirmed by longer follow-up.
